# Deep learning for differentiation of osteolytic osteosarcoma and giant cell tumor around the knee joint on radiographs: a multicenter study

**DOI:** 10.1186/s13244-024-01610-1

**Published:** 2024-02-07

**Authors:** Jingjing Shao, Hongxin Lin, Lei Ding, Bing Li, Danyang Xu, Yang Sun, Tianming Guan, Haiyang Dai, Ruihao Liu, Demao Deng, Bingsheng Huang, Shiting Feng, Xianfen Diao, Zhenhua Gao

**Affiliations:** 1 Department of Radiology, The First Affiliated Hospital, Sun Yat-Sen University, Guangzhou, Guangdong China; 2 Medical AI Lab, School of Biomedical Engineering, Health Science Centre, Shenzhen University, Shenzhen, Guangdong China; 3 National-Regional Key Technology Engineering Laboratory for Medical Ultrasound, Guangdong Key Laboratory for Biomedical Measurements and Ultrasound Imaging, School of Medicine, Shenzhen University, Shenzhen, Guangdong China; 4 Department of Radiology, Foshan Hospital of Traditional Chinese Medicine, Foshan, Guangdong China; 5 Department of Radiology, Hui Ya Hospital of The First Affiliated Hospital, Sun Yat-Sen University, Huizhou, Guangdong China; 6 Department of Radiology, People’s Hospital of Huizhou City Center, Huizhou, Guangdong China; 7 Department of Radiology, The People’s Hospital of Guangxi Zhuang Autonomous Region, Guanxi Academy of Medical Science, Nanning, Guangxi China

**Keywords:** Bone neoplasms, Knee joint, Deep learning, Radiography

## Abstract

**Objectives:**

To develop a deep learning (DL) model for differentiating between osteolytic osteosarcoma (OS) and giant cell tumor (GCT) on radiographs.

**Methods:**

Patients with osteolytic OS and GCT proven by postoperative pathology were retrospectively recruited from four centers (center A, training and internal testing; centers B, C, and D, external testing). Sixteen radiologists with different experiences in musculoskeletal imaging diagnosis were divided into three groups and participated with or without the DL model’s assistance. DL model was generated using EfficientNet-B6 architecture, and the clinical model was trained using clinical variables. The performance of various models was compared using McNemar’s test.

**Results:**

Three hundred thirty-three patients were included (mean age, 27 years ± 12 [SD]; 186 men). Compared to the clinical model, the DL model achieved a higher area under the curve (AUC) in both the internal (0.97 vs. 0.77, *p* = 0.008) and external test set (0.97 vs. 0.64, *p* < 0.001). In the total test set (including the internal and external test sets), the DL model achieved higher accuracy than the junior expert committee (93.1% vs. 72.4%; *p* < 0.001) and was comparable to the intermediate and senior expert committee (93.1% vs. 88.8%, *p* = 0.25; 87.1%, *p* = 0.35). With DL model assistance, the accuracy of the junior expert committee was improved from 72.4% to 91.4% (*p* = 0.051).

**Conclusion:**

The DL model accurately distinguished osteolytic OS and GCT with better performance than the junior radiologists, whose own diagnostic performances were significantly improved with the aid of the model, indicating the potential for the differential diagnosis of the two bone tumors on radiographs.

**Critical relevance statement:**

The deep learning model can accurately distinguish osteolytic osteosarcoma and giant cell tumor on radiographs, which may help radiologists improve the diagnostic accuracy of two types of tumors.

**Key points:**

• The DL model shows robust performance in distinguishing osteolytic osteosarcoma and giant cell tumor.

• The diagnosis performance of the DL model is better than junior radiologists’.

• The DL model shows potential for differentiating osteolytic osteosarcoma and giant cell tumor.

**Graphical Abstract:**

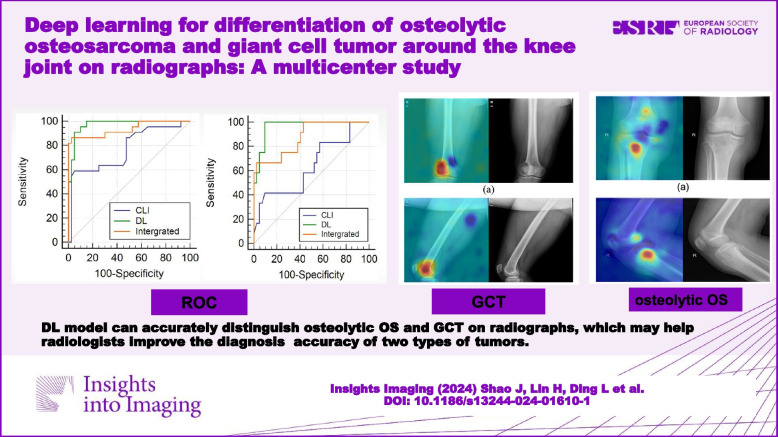

**Supplementary Information:**

The online version contains supplementary material available at 10.1186/s13244-024-01610-1.

## Introduction

Bone tumors are a group of primary or secondary neoplastic lesions of bone with various pathological types and biological behaviors [1]. According to the 5th edition of the World Health Organization (WHO) classification of bone tumors published in 2020, bone tumors are classified as benign, intermediate, or malignant [2]. Primary bone tumors of the extremities are commonly found in the bones around the knee joint [3–5], with the most common types including osteochondroma, osteosarcoma (OS), and giant cell tumor (GCT) of bone [6–8]. The imaging manifestation of osteochondroma is a benign bone tumor growing outside the bone, with diagnostic characteristics including cartilage cap coverage, and it is therefore not difficult to make a correct diagnosis according to medical imaging [9, 10]. OS and GCT of bone both show strong local aggressiveness on imaging [7, 8], but they are classified as malignant bone tumors and intermediate bone tumors according to the WHO classification of bone tumors, and their clinical treatment plans and prognosis are substantially different. Therefore, it is necessary to obtain an accurate differential diagnosis between OS and GCT before clinical treatment [1, 7, 8].

Digital radiography is widely acknowledged as the primary imaging method for diagnosing bone lesions and is extensively utilized in clinical practice [11]. Radiographs can display the overall image of bone tumors as a whole and reflect their biological behaviors, providing diagnostic and differential diagnostic information [12]. Tumoral bone formation on radiographs is a characteristic feature in the diagnosis of OS [11]. OS can be categorized into osteolytic, osteoblastic, and mixed subtypes according to the osteogenic quantity within the OS on radiographs [13]. However, distinguishing osteolytic OS from GCT becomes more challenging when tumoral bone formation is absent, especially for junior radiologist, as both exhibit localized, aggressive bone destruction [14]. Patient age holds diagnostic value in distinguishing between the two tumor types, but similar ages between patients with these two different tumors may confuse the radiologist’s judgment [11]. Therefore, we constructed a clinical model to investigate the role of clinical variables.

As an emerging machine learning technology, deep learning (DL) has been widely applied to medical image analysis of bone tumors [15–17], which can distinguish benign and malignant bone tumors [11, 12, 14, 18–21]. A recent study has highlighted DL’s potential in effectively classifying healthy and pathological X-rays in children [22]. However, previous studies have primarily focused on the benign and malignant classification of various bone tumors at different sites throughout the body [15, 18, 21], rather than specifically addressing the differential diagnosis of osteolytic OS and GCT around the knee joint. To our knowledge, there have been no reported studies utilizing DL for this specific purpose.

The purpose of this study was to develop a DL model for the differential diagnosis of osteolytic OS and GCT of bone on knee radiographs and to compare its diagnostic performance with that of radiologists with and without model assistance.

## Materials and methods

### Subjects

The retrospective study adhered to the principles outlined in the Helsinki Declaration and received institutional review board approval, including a waiver for written informed consent. This multicenter study collected patients with OS and GCT of bone around the knee joint obtained from four tertiary referral centers, from 2013 to 2022. The training set and internal test set were obtained from the First Affiliated Hospital of Sun Yat-Sen University (center A), while the external test set was obtained from Foshan Hospital of Traditional Chinese Medicine (center B), People’s Hospital of Huizhou City Center (center C) and the People’s Hospital of Guangxi Zhuang Autonomous Region (center D). Radiographs were obtained from different digital X-ray imaging devices with automatic examination parameters set in each of the four centers. Detailed information on the digital X-ray imaging devices is provided in Supplementary Table S5.

**Fig. 1:**
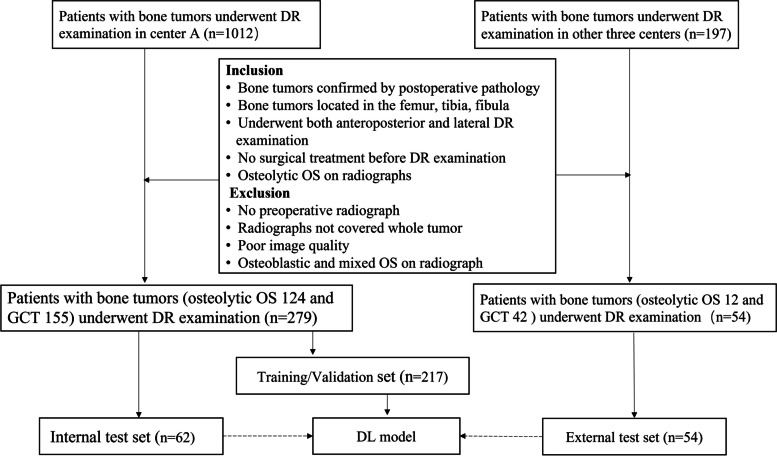
Inclusion and exclusion criteria. DR, digital radiography; OS, osteosarcoma; GCT, giant cell tumor; DL, deep learning

There were 333 patients (osteolytic OS:136, GCT:197) in this study. The inclusion and exclusion criteria are shown in Fig. 1. The osteosarcoma, in which tumoral bone formation is not observed on both anteroposterior and lateral radiographs, is considered as osteolytic osteosarcoma. According to the inclusion and exclusion criteria (Fig. 1), two radiologists (both unknown to the study, with more than 10 years of experience in reading musculoskeletal radiographs) independently reviewed all radiographs and selected the patient included in the study. The clinical data of all patients are summarized in Table 1. All included bone tumors were pathologically confirmed. Both anteroposterior and lateral radiographs were available for each patient.

**Table 1:** Demographic data of patients with osteolytic OS and GCT from four centers

Characteristics	Overall (333 patients)	Training set	Internal test set	External test set			
		Center A (217 patients)	Center A (62 patients)	Center B (24 patients)	Center C (9 patients)	Center D (21 patients)	All (54 patients)
Age (years ± SD)	27 (12)	25 (13)	26 (11)	29 (12)	34 (8)	36 (12)	33 (11)
Sex (female)	147 (44.1%)	100 (46.1%)	27 (43.6%)	12 (50.0%)	1 (11.1%)	8 (38.1%)	21 (38.8%)
Tumor type							
Osteolytic OS	136 (40.8%)	102 (47.0%)	22 (35.5%)	6 (25.0%)	0 (0%)	6 (28.6%)	12 (22.2%)
GCT	197 (59.2%)	115 (53.0%)	40 (64.5%)	18 (75.0%)	9 (100%)	15 (71.4%)	42 (77.8%)
Tumor site							
Distal femur	186 (55.9%)	125 (57.6%)	34 (54.8%)	13 (54.2%)	7 (77.8%)	7 (33.3%)	27 (50.0%)
Proximal tibia	128 (38.4%)	78 (35.9%)	26 (41.9%)	10 (41.7%)	2 (22.2%)	12 (57.1%)	24 (44.4%)
Proximal fibula	19 (5.7%)	14 (6.5%)	2 (3.2%)	1 (4.2%)	0 (0%)	2 (9.5%)	3 (5.6%)

Data shown are numbers of patients with percentages

*Abbreviations*: *Osteolytic OS* Osteolytic osteosarcoma, *GCT* Giant cell tumor

### Radiologists’ interpretations

In the internal and external test sets, sixteen radiologists from 6 tertiary referral hospitals were selected using the Multi-Reader Multi-Case (MRMC) reading method [23, 24] and Digital Imaging and Communication in Medicine (DICOM) Viewer 2.2.9 software (Medical Company, Poland) to independently evaluate the radiographs. All observers were blinded to the pathologic diagnosis results. The time taken by radiologists to evaluate radiographs was recorded.

All radiologists were categorized into three “expert committee” groups (expert decision by majority rule) based on their experience in reading musculoskeletal radiographs. There were 5 radiologists (> 2 but ≤ 5 years) in group A, 7 radiologists (> 5 but ≤ 8 years) in group B, and 4 radiologists (> 8 but < 13 years) in group C. All radiologists participated in the evaluation of performance with and without assistance from the DL model. The washout period between the two evaluations was more than 4 weeks (4–6 weeks, with an average of 5.8 weeks).

### Preprocessing

All images were downloaded from the picture archiving and communication system in DICOM format at their original dimensions and resolution, at which point the basic information of the patients on the images was removed. All images were converted from DICOM to 8-bit JPEG using MicroDicom software (Version 3.8.1.422, MicroDicom Ltd, Bulgarian). To facilitate input to the neural networks, the pixel size of each image was adjusted to 1080 by 1080, and the pixel values were scaled into the range [0,1].

### DL model building

Model training was performed in Python 3.6 (https://www.python.org) and PyTorch 1.6.0 (https://pytorch.org). We adopted the EfficientNet-B6 architecture [25] with weights pretrained on ImageNet. Five-fold cross-validation was used for model training and selecting. The model took preprocessed images with a resolution of 1080 × 1080 as inputs and output the predicted probability for each image. It employed Binary Cross Entropy (BCE) loss as the loss function and incorporated data augmentation operations such as horizontal/vertical flips, rotation, and contrast adjustments. sThe network was optimized with the following parameters: basic learning rate = 1 × 10^-4^; batch size = 2; and epoch = 150. The model with the minimum validation loss was selected for testing on both the internal and external test sets. 

### Clinical model and integrated model building

A clinical model based on clinical variables, including patient age, sex and tumor site (distal femur, proximal tibia and proximal fibula), was developed using logistic regression. Sex was coded using binary coding, and the tumor site was coded using one-hot coding. The clinical model was trained using a cross-validation strategy. The model with the best validation performance was selected for testing on both the internal and external test sets. Additionally, we incorporated the predictions generated by the DL model as a variable into the clinical model to establish an integrated model. Similarly, the training and testing strategy for the integrated model remains consistent with that of the clinical model.

### Feature visualization and analysis

For feature visualization, gradient-weighted class activation mapping (Grad-CAM) [26] was used to generate the Grad-CAM maps with the last convolution layer of the DL model, representing the model’s attention to different portions of an input radiograph. For the feature analysis, the t-distributed stochastic neighbor embedding (t-SNE) [27] algorithm was used to reduce the 2304-dimensional features extracted by the DL model to three dimensions and then visualize them in 3D space to display the difference in the distribution of the features of GCT and OS.

### Statistical analysis

In comparisons of clinical variables between two groups and to assess the impact of different digital X-ray imaging devices on DL model performance, the chi-square test was used for categorical variables (sex, tumor site, digital X-ray imaging device), and the independent samples *t* test was used for continuous variables (age). Additionally, we grouped patients according to patient age and divided into quartiles to analyze diagnostic performance of different radiologists in different age groups. The accuracy, sensitivity, specificity, area under the ROC curve (AUC), and corresponding 95% confidence interval were calculated. The optimal cutoff value was determined by the maximum Youden index. The ROC curves of various models were compared using the DeLong test. The accuracy of the various models was compared using McNemar’s test. All analyses were conducted using R (Version 4.0.4), SPSS (Version 24.0), and MedCalc (Version 15.8) statistical software. *p* < 0.05 was considered statistically significant.

## Results

### Patient characteristics

A total of 333 patients with bone tumors were included in this study, with an average age of 27 years, including 186 males and 147 females. Among them, there were 136 patients with osteolytic OS (male: 72, female: 64), aged 3–61 years, with an average age of 19 years, and 197 patients with GCT of bone (male: 117, female: 80), aged 12–70 years, with an average age of 32 years (as shown in Table 1). There was no significant difference in sex between patients with osteolytic OS and GCT; however, there was a significant difference in age and tumor site between the two types of bone tumors, as shown in Table S1.

### Model performance

**Fig. 2:**
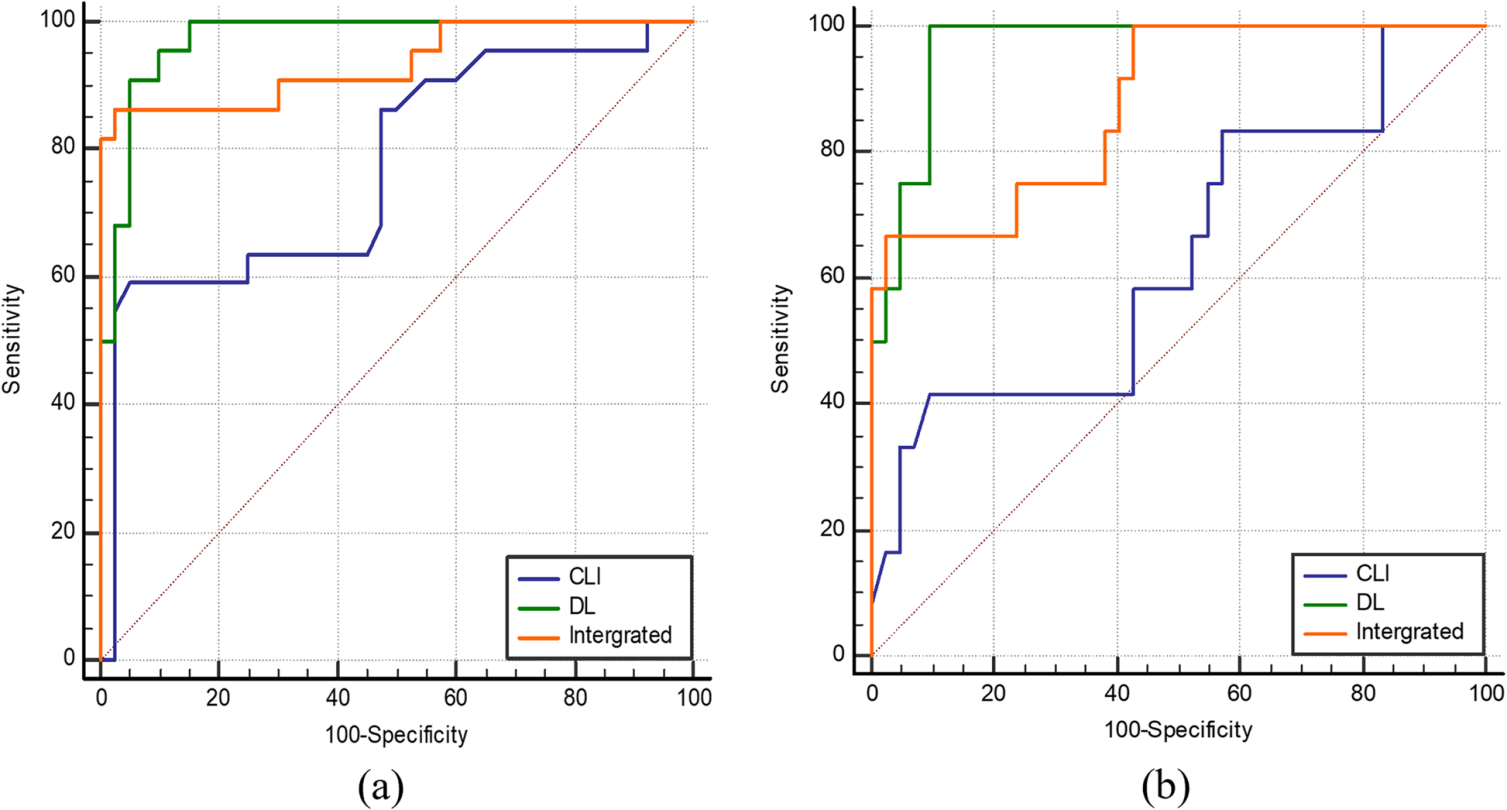
ROC curves of the three models in the internal test set (**a**) and in the external test set (**b**). CLI, clinical model; DL, DL model

The DL model achieved an AUC of 0.94 (0.90–0.97) in the training set, 0.97 (0.90–1.00) in the internal test set, and 0.97 (0.88–1.0) in the external test set (all *p* < 0.001; as shown in Table 2, Fig. 2a). According to the DeLong test, we found no evidence of a significant difference in the performance of the DL model between the internal test set and external test set (*p* = 0.79). Additionally, there was no significant difference in the DL model performance among digital X-ray imaging devices (*p* = 0.43) according to the chi-square test (as shown in Table S5).

**Table 2:** Diagnostic performance of the deep learning model, clinical model, and integrated model in the training set, internal test set, and external test set

Dataset	AUC (95% CI)	Accuracy	Sensitivity	Specificity	*p* value
**Training set (*n* = 217)**					
DL model	0.94 (0.90–0.97)	91.2% (198/217)	90.2% (92/102)	92.2% (106/115)	< 0.001
**Internal test set (*n* = 62)**					
DL model	0.97 (0.90–1.00)	93.5% (58/62)	90.8% (20/22)	95.0% (38/40)	< 0.001
Clinical model	0.77 (0.65–0.87)	82.3% (51/62)	59.1% (13/22)	95.0% (38/40)	< 0.001
Integrated model	0.94 (0.84–0.98)	93.5% (58/62)	86.4% (19/22)	97.5% (39/40)	< 0.001
**External test set (*n* = 54)**					
DL model	0.97 (0.88–1.00)	92.6% (50/54)	100% (12/12)	90.5% (38/42)	< 0.001
Clinical model	0.64 (0.50–0.76)	79.6% (43/54)	41.7% (5/12)	90.5% (38/42)	0.17
Integrated model	0.88 (0.76–0.95)	90.7% (49/54)	66.7% (8/12)	97.6% (41/42)	< 0.001
**Total test set (*n* = 116)**					
DL model	0.97 (0.92–1.00)	93.1% (108/116)	94.1% (32/34)	92.7% (76/82)	< 0.001
Clinical model	0.72 (0.63–0.80)	81.0% (94/116)	52.9% (18/34)	92.7% (76/82)	< 0.001
Integrated model	0.91 (0.85–0.96)	92.2% (107/116)	79.4% (27/34)	97.6% (80/82)	< 0.001

*p* value represents a comparison between the AUC value of the model and chance (AUC = 0.5)

*DL* Deep learning, integrated model indicates DL model combined with clinical model

The logistic regression clinical model based on clinical variables achieved an AUC of 0.77 (0.65–0.87, *p* = 0.001) in the internal test set and 0.64 (0.50–0.76, *p* = 0.17) in the external test set (as shown in Table 2, Fig. 2b).

According to the DeLong test, the diagnostic performance of the DL model was superior to that of the clinical model in both the internal (*p* = 0.008) and external test sets (*p* < 0.001) and superior to that of the integrated model in both the internal (*p* = 0.28) and external test sets (*p* = 0.11).

### Diagnostic performance of radiologists’ evaluation with and without the DL model

The comparative results of the diagnostic performance among the DL model, radiologists without model assistance, and radiologists with model assistance are shown in Table 3, S2, S3, and S4. In the total test set composed of the internal and external test sets, there was a significant difference in the diagnostic performance between the DL model and the expert committee “A” (junior radiologists) (*p* < 0.001). The diagnostic accuracy of the expert committee “A” was significantly improved with the help of the DL model (*p* = 0.05). Specifically, among the five junior radiologists (> 2 but ≤ 5 years), the accuracy of the DL model was better than that of the radiologists (93.1% vs. 52.6%, 55.2%, 81.0%, 75.0%, 78.5%; all *p* < 0.05), and the accuracy of all the junior radiologists was improved with the aid of the model (52.6% to 86.2%, *p* < 0.001; 55.2% to 87.9%, *p* < 0.001; 81.0% to 90.5%, *p* = 0.49; 75.0% to 80.2%, *p* = 0.35; 78.5% to 92.2%, *p* = 0.003; as shown in Table S2). Additionally, the diagnostic performance of the DL model was superior to that of the expert committee “C” (senior radiologists), and the diagnostic accuracy of the expert committee “C” was improved with the help of the DL model but the difference was insignificant. Specifically, among the four senior radiologists (> 8 years), the accuracy of the DL model was better than that of the radiologists (93.1% vs. 85.3%, 85.3%, 88.8%, 86.2%; *p* = 0.06, *p* = 0.06, *p* = 0.25, *p* = 0.09), and the accuracy of three of the senior radiologists was improved with the aid of the model (85.3% to 87.1%, *p* = 0.70; 85.3% to 90.5%, *p* = 0.23; 88.8% to 92.2%, *p* = 0.37; as shown in Table S4).

**Table 3:** Diagnostic performance comparison among the DL model, radiologist evaluation without model assistance, and radiologist evaluation with model assistance

Model	ACC (95% CI)	*p* value
Deep learning	93.1 (87.0–96.5) [108/116]	
Expert committee A-Wt	72.4 (63.7–0.79.7) [84/116]	< 0.001
Expert committee A-Wi	91.4 **(**84.9–0.95.3) [106/116]	0.051*
Expert committee B-Wt	88.8 (81.8–93.3) [103/116]	0.25
Expert committee B-Wi	90.5 (83.8–94.6) [105/116]	0.67*
Expert committee C-Wt	87.1 (79.8–92.0) [101/116]	0.32
Expert committee C-Wi	89.7 (82.8–94.0) [104/116]	0.54*

Expert committees A, B, and C indicated three groups of radiologists with different levels of experience in reading musculoskeletal radiographs. “Wt” means without DL model assistance, “Wi” means with DL model assistance, and the *p* value reflects the comparison of accuracy between different pairs of models, indicating “Wt” versus DL and “Wt” versus “Wi” (indicated by *) (the same below)

Among 16 radiologists in reading radiographs from 116 patients, 9 had shorter diagnostic time with DL assistance (decrease of 1 to 15 min), while 4 had longer diagnostic time (increase of 5 to 10 min) and 3 remained unchanged (Tables S2–S4). Patients in the total test set were further grouped according to patient age, divided into quartiles: 27 patients (OS 21 and GCT 4) in group A; 30 patients (OS 4 and GCT 26) in group B; 31 patients (OS 1 and GCT 30) in group C; and 28 patients (OS 7 and GCT 22) in group D. The statistical results are shown in Table 4. In the 21–30-year age group, the diagnostic performance of the DL model was better than that of the junior radiologist group (*p* = 0.003).

**Table 4:** Comparison of diagnostic performance between the DL model and radiologist evaluation in the patient subgroups

Age	Model	Accuracy 95% CI)	*p* value
Age (< 21 years, *n* = 27)	DL	100 [27/27]	
	Expert committee -A	88.9 (71.9–96.2) [24/27]	0.25
	Expert committee -B	96.3 (81.7–99.3) [26/27]	0.99
	Expert committee	96.3 (81.7–99.3) [26/27]	0.99
Age (21–30 years, *n* = 30)	DL	96.7 (83.3–99.4) [29/30]	
	Expert committee -A	60.0 (42.3–75.4) [18/30]	0.003
	Expert committee -B	83.3 (66.4–92.7) [25/30]	0.22
	Expert committee -C	86.8 (70.3–94.7) [26/30]	0.38
Age (30–38 years, *n* = 31)	DL	90.3 (75.1–96.7) [28/31]	
	Expert committee -A	74.2 (56.8–86.3) [23/31]	0.13
	Expert committee -B	83.9 (67.4–92.9) [26/31]	0.63
	Expert committee -C	83.9 (67.4–92.9) [26/31]	0.63
Age (≥ 38 years, *n* = 28)	DL	85.7 (68.5–94.3) [24/28]	
	Expert committee -A	85.7 (68.5–94.3) [24/28]	0.99
	Expert committee -B	92.9 (77.4–98.0) [26/28]	0.63
	Expert committee -C	92.9 (77.4–98.0) [26/28]	0.63

*p* value indicates significant differences in accuracy between expert committee and DL

### Feature visualization and analysis

**Fig. 3:**
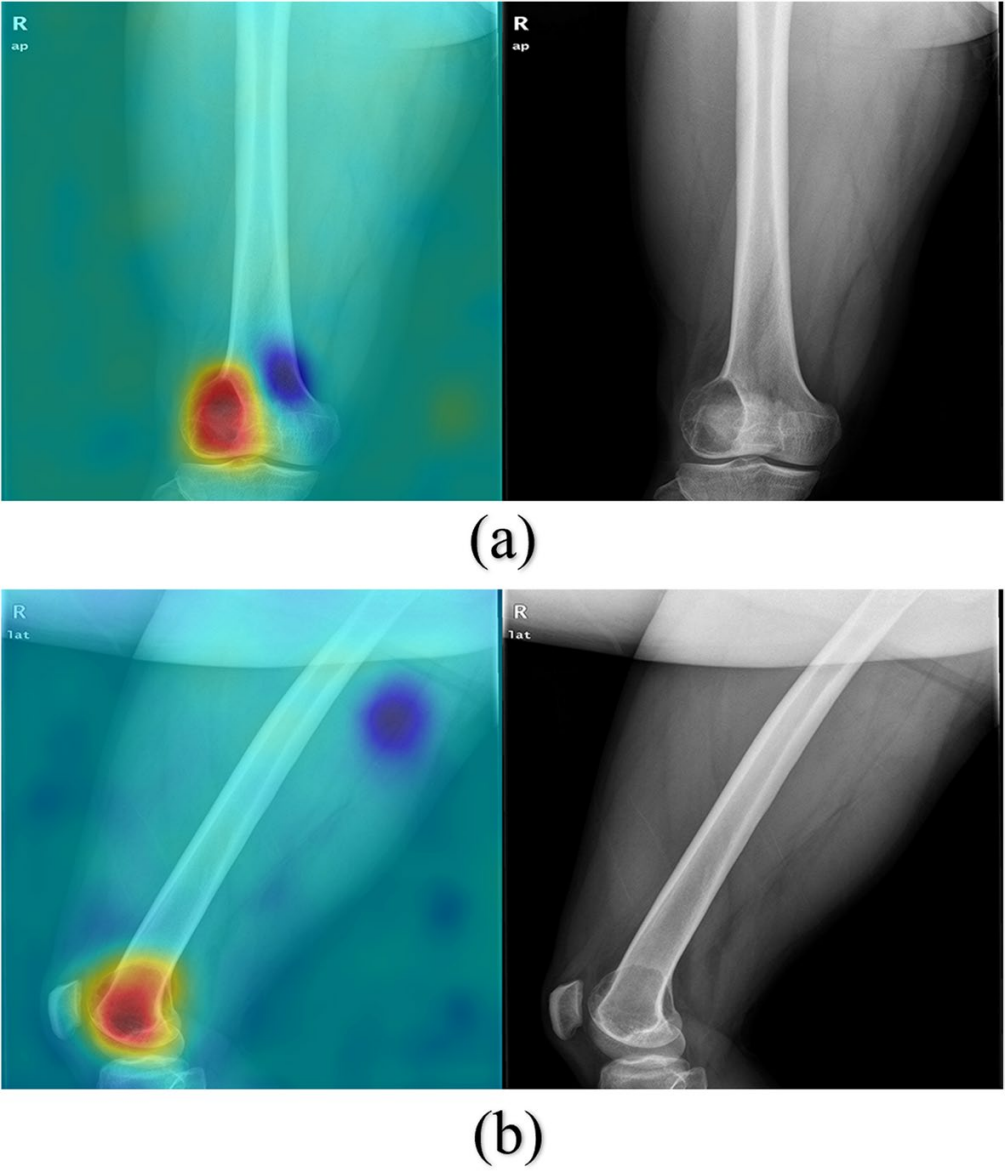
Anteroposterior (**a**) and lateral (**b**) radiographs of GCT of the distal femur and their overlapping Grad-CAM maps

**Fig. 4:**
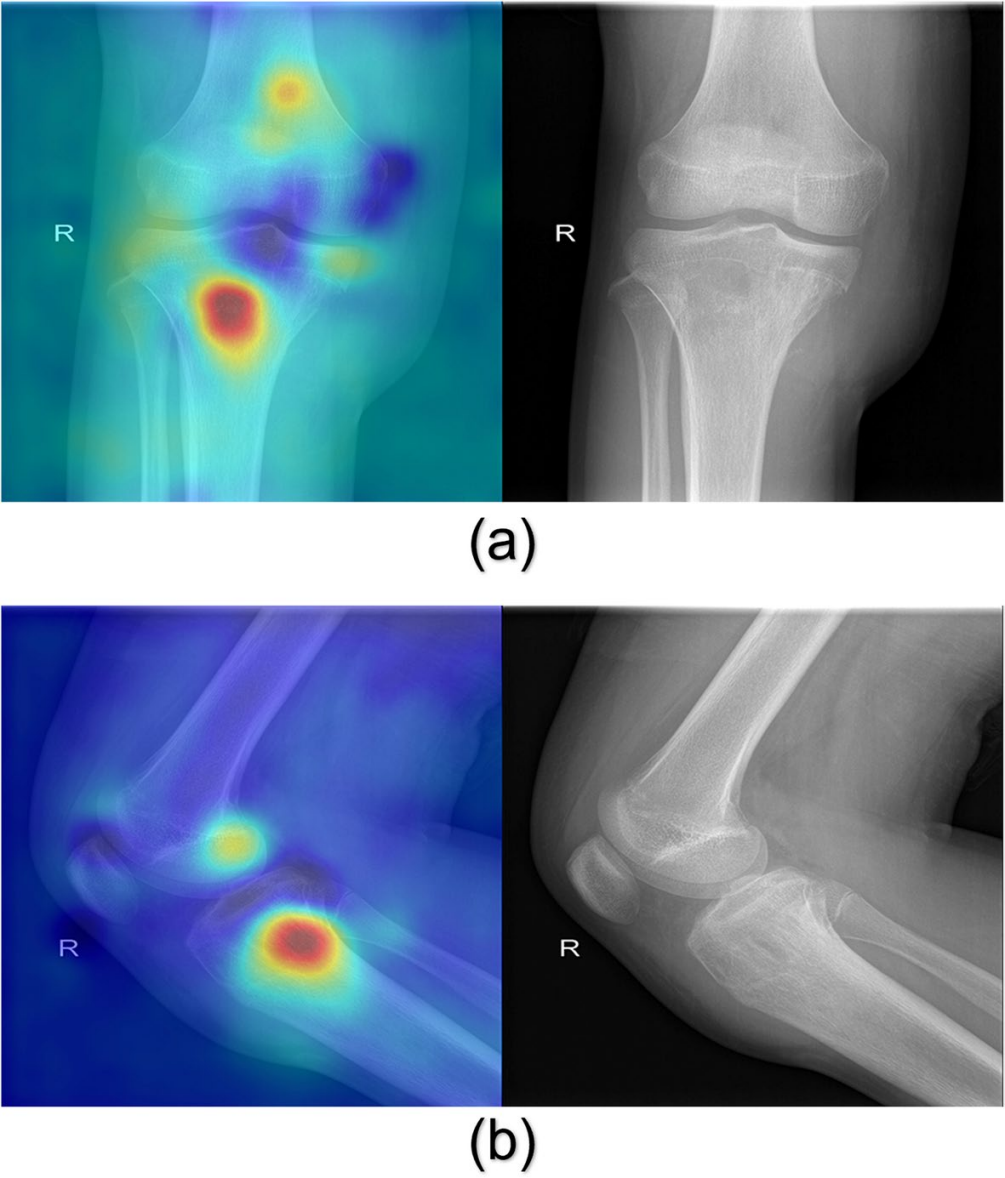
Anteroposterior (**a**) and lateral (**b**) radiographs of OS of the proximal tibia and their overlapping Grad-CAM maps

For the 116 patients in the total test set, the main features of the DL model concentrated in the area of bone destruction caused by the tumor on the radiographs and their corresponding overlapping Grad-CAM heatmaps. The red areas in the heatmaps were simultaneously present in 95 patients’ bone tumor areas on the anteroposterior and lateral radiographs (shown in Figs. 3 and 4), but only in 19 patients’ bone tumor areas on the anteroposterior radiographs and 2 patients’ bone tumor areas on the lateral radiographs.

**Fig. 5:**
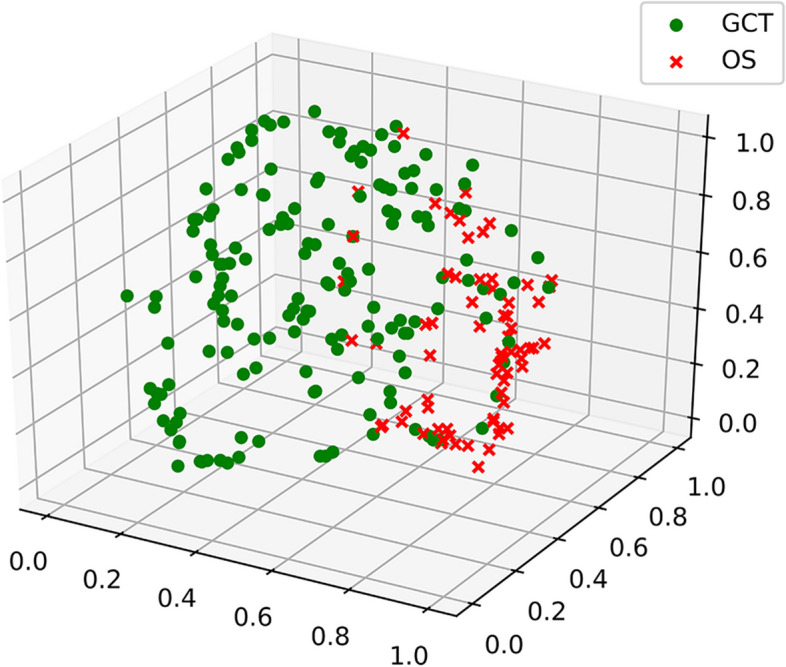
3D display of features extracted by the DL model in the total test set: red crosses represent osteolytic OS patients; green dots represent GCT patients

The t-SNE feature analysis results from the total test set showed that the spatial distribution of extracted image features for GCT and OS were different (shown in Fig. 5), indicating that the spatial distribution of the features extracted by the DL model was involved in the differential diagnosis of the two diseases.

## Discussion

In this multicenter study, DL models were developed and validated to distinguish between osteolytic OS and GCT on radiographs and compared to radiologists. Overall, the DL model showed a higher diagnostic accuracy than the clinical model or integrated model (0.97 vs. 0.72, 0.91). In the test set, the DL model achieved higher accuracy than the junior expert committee (93.1% vs. 72.4%; *p* < 0.001) and was comparable to the intermediate and senior expert committee (93.1% vs. 88.8%, 87.1%; *p* = 0.25, *p* = 0.35, respectively). With DL model assistance, the performance of junior expert committee improved (72.4% to 91.4%, *p* = 0.051).

This study revealed a significant difference in age between the two types of bone tumors, which is consistent with previous literature, indicating that patient age holds some diagnostic value in distinguishing between the two tumor types [11]. Regarding the tumor site, this study revealed a statistically significant difference between osteolytic OS and GCT, which is inconsistent with previous research about OS and GCT [6–8]. We believe that this may be attributed to sample bias due to the limited number of osteolytic OS patients included. The logistic regression model based on clinical characteristics exhibited poor performance in the differential diagnosis of the two tumor sites in the overall test set (AUC = 0.72).

In clinical practice, radiologists may misdiagnose osteolytic OS as GCT of bone and vice versa due to the similar appearances on radiographs. The diagnostic accuracy of radiologists in distinguishing between osteolytic OS and GCT of bone is greatly influenced by their clinical experience in reading musculoskeletal radiographs (accuracy ranging from 52.6% to 90.5%, Tables S2, S3, S4). This study used the MRMC method and voting rules (minority follows majority) to demonstrate that radiologists with different levels of clinical experience exhibit different diagnostic performances (accuracy: 76.7%, 88.8%, and 87.1%). Greater clinical experience in reading musculoskeletal radiographs led to improved accuracy of the radiologists in differentiating between osteolytic OS and GCT of bone, which aligns with the learning growth curve characteristics [7]. We conducted a stratified analysis of patients with either type of tumor at different ages. The study revealed that within the patient group with age range of 21 to 30 years, the radiologists’ diagnostic accuracy was lower, while the DL model demonstrated significantly higher accuracy (Table 4). This indicates that when faced with patients of similar age presenting with these aggressive bone tumors, radiologists may experience reduced diagnostic accuracy due to the similarities in radiographic appearance. When 16 radiologists evaluated all radiographs with AI, the evaluation time varied compared to that without AI assistance (Tables S2–S4), which may be due to the different experiences. Of these, 11 radiologists’ diagnostic accuracy with AI was improved, suggesting that artificial intelligence can provide valuable information. Our study demonstrated that the DL model outperformed both the clinical model and the combined model. This suggests that although patient age has a certain reference value in the differential diagnosis between tumor types, the overlap among the patient ages may have reduced the diagnostic accuracy. In contrast, the radiographic appearance can reflect the underlying pathology and biological behavior of the bone tumors, highlighting the superior diagnostic value of the DL model based on radiograph features.

Our proposed DL model identified the tumor area and its surrounding normal bone in the full field of the radiograph, rather than only choosing the tumor area [18, 21], avoiding the need for tumor area labeling. Additionally, the DL model takes into account not only the bone tumor itself but also the shape and size of the bone in which the tumor is located, thereby capturing important variations associated with age. The DL model proposed in this study may include clinical information about age and tumor site, resulting in better diagnostic performance. According to the Grad-CAM heatmaps and t-SNE feature analysis, the features extracted by the DL model concentrated in the bone destruction area caused by the tumors and were spatially able to differentiate between the two types of tumors to some extent.

This study was subject to several limitations. First, the research only focused on two primary aggressive bone tumors around the knee joint, OS and GCT of bone, without considering other bone tumors. In our opinion, the region around the knee joint is the most common site for primary bone tumors, and the most common aggressive bone tumors among them are OS and GCT of bone [3–8]. The radiographic manifestations of osteolytic OS and GCT are similar, which can easily confuse the diagnosis [10]. In the future, we intend to incorporate other types of bone tumors to expand the application scope of our model in real clinical scenarios. Second, this study only utilized radiographic information for the establishment of the DL model without incorporating other imaging modalities, such as CT or MRI. This is because digital radiography is recognized as a first-line imaging modality for bone lesion evaluation [11], facilitating the widespread implementation of the DL model in clinical practice. Third, we used the MRMC method to evaluate the radiologist’s diagnostic performance, but the sample size of the patients in the external test set was limited. To further evaluate the DL model, we may continue to collect more data in future studies.

In conclusion, we developed a DL model for the differential diagnosis of osteolytic OS and GCT of bone on knee radiographs. Our model outperformed junior radiologists in terms of diagnostic accuracy, required less time, and enhanced the diagnostic performance of radiologists as an assistive tool, which demonstrated its potential for accurate differential diagnosis in clinical applications.

### Supplementary Information

**Additional file 1: Table S1.** Comparison of the clinical characteristics of the patients with osteolytic OS and GCT. **Table S2.** Diagnostic performance comparison among the DL model, radiologist’s evaluation without model assistance, and radiologist’s evaluation with model assistance in group A. **Table S3.** Diagnostic performance comparison of the DL model, radiologist’s evaluation without model assistance, and radiologist’s evaluation with model assistance in group B. **Table S4.** Diagnostic performance comparison of the DL model, radiologist’s evaluation without model assistance, and radiologist’s evaluation with model assistance in group C. **Table S5.** Detailed information on digital X-ray imaging devices.

## Data Availability

The data underlying this article will be shared on reasonable request to the corresponding author.

## Abbreviations

DL: Deep learning
GCT: Giant cell tumor
OS: Osteosarcoma
