# Supplementary material for: Deep learning for differentiation of osteolytic osteosarcoma and giant cell tumor around the knee joint on radiographs: a multicenter study
Source: Insights Imaging. 2024 Feb 7;15:35. doi: 10.1186/s13244-024-01610-1 (PMC10847082; doi:10.1186/s13244-024-01610-1)
Supplement: Supplementary file 1 — Additional file 1: Table S1. Comparison of the clinical characteristics of the patients with osteolytic OS and GCT. Table S2. Diagnostic performance comparison among the DL model, radiologist’s evaluation without model assistance, and radiologist’s evaluation with model assistance in group A. Table S3. Diagnostic performance comparison of the DL model, radiologist’s evaluation without model assistance, and radiologist’s evaluation with model assistance in group B. Table S4. Diagnostic performance comparison of the DL model, radiologist’s evaluation without model assistance, and radiologist’s evaluation with model assistance in group C. Table S5. Detailed information on digital X-ray imaging devices. [file 13244_2024_1610_MOESM1_ESM.docx]

**Deep learning for differentiation of osteolytic osteosarcoma and giant cell tumor around the knee joint on radiographs:**

**ELECTRONIC SUPPLEMENTARY MATERIAL**

**Table S1**: Comparison of the clinical characteristics of the patients with osteolytic OS and GCT

| Characteristics | Osteolytic OS  (136) | | GCT  (197) | Statistical method | p value |
| --- | --- | --- | --- | --- | --- |
| Age (years ± SD) | | 19(10) | 32(11) | Independent Sample t Test | 0.002 |
| Sex (Female) | | 64(47.2%) | 80(40.4%) | Chi-Square Test | 0.27 |
| Tumor Site | |  |  | Chi-Square Test | 0.03 |
| Distal femur | | 91(66.9%) | 95(48.2%) |  |  |
| Proximal tibia | | 39(28.7%) | 89(45.2%) |  |  |
| Proximal fibula | | 6(4.4%) | 13(6.6%) |  |  |

Note: P value was derived from independent samples t-test or chi-square test between patients with osteolytic OS and GCT.

**Table S2**: Diagnostic performance comparison among the DL model, radiologist’s evaluation without model assistance, and radiologist’s evaluation with model assistance in group A

| Model | Accuracy (95% CI) | P value | Time |
| --- | --- | --- | --- |
| Deep Learning | 93.1(87.0-96.5) [108/116] |  | 5.5 min |
| Radiologist A1-Wt | 52.6(43.6-61.5) [61/116] | <0.001 | 45 min |
| A1-Wi | 86.2**(**78.8-91.3) [100/116] | <0.001* | 40min |
| Radiologist A2-Wt | 55.2(46.1-63.9) [64/116] | <0.001 | 46 min |
| A2-Wi | 87.9(87.8-92.7) [102/116] | <0.001* | 42min |
| Radiologist A3-Wt | 81.0(73.0-87.1) [94/116] | 0.007 | 43 min |
| A3-Wi | 90.5(83.8-94.9) [105/116] | 0.49* | 33min |
| Radiologist A4-Wt | 75.0(66.4-82.0) [87/116] | <0.001 | 65 min |
| A4-Wi | 80.2(72.0-86.4) [93/116] | 0.35* | 70min |
| Radiologist A5-Wt | 78.5(70.1-84.9) [91/116] | 0.002 | 70 min |
| A5-Wi | 92.2(85.9-95.9) [98/116] | 0.003* | 57min |

Note: A1-A5 indicate 5 doctors with 2-5 years of experience in reading musculoskeletal radiographs, “Wt” means without DL model assistance, “Wi” means with DL model assistance, p value reflects a comparison of the accuracy between different models, indicating “Wt” versus DL and “Wt” versus “Wi” (indicated by * sign) (the same below).

**Table S3**: Diagnostic performance comparison of the DL model, radiologist’s evaluation without model assistance, and radiologist’s evaluation with model assistance in group B

|  | | Accuracy (95% CI) | | P value | | Time |
| --- | --- | --- | --- | --- | --- | --- |
| Deep Learning | | 93.1(87.0-96.5) [108/116] | |  | | 5.5 min |
| Radiologist B1-Wt | | 88.8(81.8-93.3) [103/116] | | 0.26 | | 40 min |
| B1-Wi | | 89.6(82.8-94.0) [104/116] | | 0.85* | | 37min |
| Radiologist B2-Wt | | 79.3(71.1-85.7) [92/116] | | 0.002 | | 49 min |
| B2-Wi | | 72.4(63.2-80.1) [84/116] | | 0.22* | | 48min |
| Radiologist B3-Wt | | 81.0(73.0-87.1) [94/116] | | 0.009 | | 69 min |
| B3-Wi | | 77.6(69.2-84.6) [90/116] | | 0.52* | | 69min |
| Radiologist B4-Wt | 81.9(73.9-87.9) [95/116] | | 0.01 | | 40 min | |
| B4-Wi | 75.9**(**67.3-82.7) [88/116] | | 0.26* | | 25min in | |
| Radiologist B5-Wt | 83.6(75.8-89.3) [97/116] | | 0.02 | | 33 min | |
| B5-Wi | 88.8(81.8-93.3) [103/116] | | 0.25* | | 25min | |
| Radiologist B6-Wt | 90.5(83.8-94.6) [105/116] | | 0.47 | | 46 min | |
| B6-Wi | 85.3(77.8-90.6) [99/116] | | 0.23* | | 56min | |
| Radiologist B7-Wt | 76.7(68.3-83.5) [89/116] | | <0.001 | | 60 min | |
| B7-Wi | 87.9(80.8-92.7) [102/116] | | 0.03* | | 65min | |

Note: B1-B7 indicate 7 doctors with 5-8 years of experience in reading musculoskeletal radiographs, “Wt” means without DL model assistance, “Wi” means with DL model

assistance, p value reflects a comparison of the accuracy between different models, indicating “Wt” versus DL and “Wt” versus “Wi” (indicated by * sign).

**Table S4**: Diagnostic performance comparison of the DL model, radiologist’s evaluation without model assistance, and radiologist’s evaluation with model assistance in group C

|  | Accuracy (95% CI) | P value | Time |
| --- | --- | --- | --- |
| Deep Learning | 93.1(87.0-965) [108/116] |  | 5.5 min |
| Radiologist C1-Wt | 85.3(77.8-90.6) [99/116] | 0.06 | 48 min |
| C1-Wi | 87.1(79.8-92.0) [101/116] | 0.70* | 57min |
| Radiologist C2-Wt | 85.3(77.8-90.6) [99/116] | 0.06 | 32 min |
| C2--Wi | 90.5(83.8-94.6) [105/116] | 0.23* | 32min |
| Radiologist C3-Wt | 88.8(81.8-93.3) [103/116] | 0.25 | 38 min |
| C3-Wi | 92.2(85.9-95.9) [107/116] | 0.37* | 24min |
| Radiologist C4-Wt | 86.2(78.8-91.3) [100/116] | 0.09 | 65 min |
| C4-Wi | 80.2(72.0-86.4) [93/116] | 0.22* | 65min |

Note: C1-C4 indicate 4 doctors with 8-13 years of experience in reading musculoskeletal radiographs, “Wt” means without DL model assistance, “Wi” means with DL model assistance, p value reflects a comparison of the accuracy between different models, indicating “Wt” versus DL and “Wt” versus “Wi” (indicated by * sign) .

**Table S5:** Detailed information on digital X-ray imaging devices

|  | **Accuracy** | **Sensitivity** | **Specificity** |
| --- | --- | --- | --- |
| **Overall** | 93.1% (108/116) | 94.1% (32/34) | 92.7% (76/82) |
| **Devices** (manufacturer information) |  |  |  |
| Digital Diagnost, Philips Medical Systems | 94.3% (33/35) | 77.8% (7/9) | 100.0% (26/26) |
| YSIO, Siemens Healthineers | 92.0% (23/25) | 100.0% (10/10) | 86.7% (13/15) |
| Definium 6000, GE Healthcare | 95.7% (22/23) | 100.0% (6/6) | 94.2% (16/17) |
| DX-D600, AFGA Medical Systems | 93.3% (14/15) | 100.0% (3/3) | 91.7% (11/12) |
| Rad Speed Plus, Shimadzu Medical Systems | 100.0% (14/14) | 100.0% (6/6) | 100.0% (8/8)- |
| DigiEye 680, ShenZhen Mindray Bio-Medical Electronics | 50.0% (2/4) |  | 50.0% (2/4) |
